# Identification of Interleukin (IL)-33 Inhibitory Constituents from *Canavalia gladiata* Pods

**DOI:** 10.3390/antiox13070767

**Published:** 2024-06-25

**Authors:** Le Ba Vinh, Seung Hyuck Shin, Yoo Kyong Han, Young Jun Kim, Nguyen Cao Cuong, Soohwan Oh, Ki Yong Lee

**Affiliations:** 1 College of Pharmacy, Korea University, Sejong 30019, Republic of Korea; vinhrooney@gmail.com (L.B.V.); tmdgur0913@naver.com (S.H.S.); yookyong05@korea.ac.kr (Y.K.H.); yjkim22@korea.ac.kr (Y.J.K.); soohwanoh@korea.ac.kr (S.O.); 2 Institute of Marine Biochemistry, Vietnam Academy of Science and Technology, Hanoi 11355, Vietnam; 3 Faculty of Medicine and Pharmacy, Yersin University, Da Lat 66100, Vietnam; nguyencaocuong2712@gmail.com

**Keywords:** *Canavalia gladiata*, sword bean pods, interleukin-33, molecular docking, molecular dynamic

## Abstract

Interleukin (IL)-33, a member of the IL-1 cytokine family, plays a vital role in immune system regulation and inflammation, with oxidative stress being implicated in its expression. During the search for compounds from natural sources with potential as therapeutic agents for allergic diseases via IL-33 signal modulation, we discovered significant IL-33 inhibitory activity in the methanol extract of *Canavalia gladiata* (sword bean) pods. Through chromatographic separation and liquid chromatography–mass spectrometry, we isolated 11 compounds (**1**–**11**) from the methanol extract. Furthermore, we assessed the inhibitory effects of these substances on IL-33/ST2 signaling in processes related to inflammatory and autoimmune diseases using an enzyme-linked immunosorbent assay. Among them, compounds **7**, **10**, and **11** exhibited substantial IL-33 inhibitory efficacy, with values reaching 78%, 86%, and 79% at 100 µM, respectively. Remarkably, compounds **7**, **10**, and **11** demonstrated significant and dose-dependent inhibition of IL-33 signaling at concentrations of 10, 50, and 100 µM. Computational molecular docking and dynamic simulations further elucidated the underlying mechanisms. These findings have promising pharmacological implications for allergy prevention and treatment associated with flavonoid glycosides derived from *C. gladiata.*

## 1. Introduction

Allergic diseases (ADs), characterized by immune-related conditions, are primarily initiated by an IgE-driven response to otherwise harmless environmental antigens, known as allergens. This hypersensitive reaction can result in a wide range of symptoms, including itching, sneezing, wheezing, and skin rashes [1]. The increasing prevalence of ADs contributes significantly to global healthcare costs. According to the World Health Organization, it is projected that by 2050, around 50% of the global population will be affected by ADs [2]. Moreover, ADs are linked to various other conditions such as cancer, inflammatory diseases, asthma, rheumatologic diseases, neurological disorders, and cardiovascular problems [3]. Therefore, a comprehensive approach using natural remedies to treat ADs is highly desirable.

Allergic reactions encompass a range of diseases, with allergic conditions arising from a complex interplay of factors including both genetic and environmental ones [4]. These interactions encompass various cell types, such as macrophages, epithelial cells, endothelial cells, dendritic cells, and mast cells. Among these, interleukin (IL)-33, a cytokine belonging to the IL-1 family, is associated with multiple cell types. Numerous reports have illustrated the associations of IL-33, thymic stromal lymphopoietin factor, and cytokines with a range of conditions, including inflammation, asthma, and atopic dermatitis [5,6,7]. IL-33, through its interaction with the ST2 receptor, can initiate T helper type 2 (Th2) immune responses, playing a role in allergic inflammation [8]. Also, oxidative stress amplifies the expression of IL-33 in human airway epithelial cells [9]. The balance between oxidative stress and antioxidant responses plays an important role in controlling IL-33 release in airway epithelium. IL-33 has been demonstrated to stimulate the production of antioxidant enzymes, including superoxide dismutase (SOD), catalase, and glutathione peroxidase (GPx), across various cell types [10]. Moreover, IL-33 has been associated with the upregulation of nuclear factor erythroid 2-related factor 2 (Nrf2), which serves as a key regulator of genes involved in antioxidant responses. Activation of the IL-33/ST2 pathway has been correlated with the mitigation of oxidative stress through augmentation of the cellular antioxidant defense system. Moreover, secondary metabolites isolated from traditional folk medicines that target IL-33 may hold significant value in the investigation of their relationship with the IL-33/ST2 signaling pathways implicated in atopic dermatitis [11].

*Canavalia gladiata* (sword bean), originally from Asia, has spread to the West Indies, Africa, South America, and Australia [12]. In Chinese herbal medicine and cuisine, various products including foods and sauces are made from *C. gladiata*, a legume that is popular in Asia and Africa [12]. Green sword bean fruit is a common domestic vegetable, and the mature seeds are used in cooking. Traditional Chinese medicine has utilized the bean to treat conditions such as purulent irritation, sinusitis, hemorrhoids, and boils. Sword beans exhibit a wide range of bioactive properties, including antioxidative, antibacterial, anti-gastric inflammatory, and antiangiogenic activities, and they have the potential to alleviate digestive disorders [13]. Interestingly, sword beans lack isoflavones, common bioactive substances found in soybeans and black beans [14]. Sword beans exhibit high levels of total phenolics and flavonoids, comparable to soybeans and black beans. To evaluate their potential as a food source, it is crucial to understand their chemical composition. Sword beans contain bioactive compounds, including gallic acid derivatives, diterpene glycosides, and flavanol glycosides [14]. Nevertheless, there has been limited research on the chemical constituents and chemical profile of the sword bean. Additionally, its potential anti-allergic effects have not been reported previously. Therefore, it is essential to explore bioactive compounds in *C. gladiata* with potential anti-allergic properties. As a continuation of our recent studies on the secondary metabolites and bioactive properties of traditional medicines, we emphasize the analytical processes, isolation, structure, and potential IL-33/ST2-inhibiting activity [15,16]. Furthermore, the underlying mechanisms of IL-33 inhibitory activity were uncovered through in vitro and in silico studies.

## 2. Materials and Methods

### 2.1. Chemicals and Reagents

One-dimensional (1D) and two-dimensional (2D) nuclear magnetic resonance data, including ^1^H (600 MHz) and ^13^C (150 MHz), were recorded using a Bruker Ultrashield 600 MHz Plus spectrometer (Bruker, Bremen, Germany). CC utilized silica gel (60F245 and RP-18 F254s; Merck, Darmstadt, Germany) and Sephadex LH-20 (Sigma-Aldrich, St. Louis, MO, USA). Thin-layer chromatography (TLC) was conducted on silica gel 60 F254. The purity of the discovered compounds was evaluated by exposure to a UV lamp emitting light at either 254 or 365 nm, followed by immediate heating after spraying with 10% H_2_SO_4_. The chemicals and solvents used in the reaction were obtained from Sigma-Aldrich/Research Organics and used as-is without further purification.

### 2.2. Liquid Chromatography Quadrupole Time-of-Flight Mass Spectrometry Conditions

We employed a previously described method to investigate the secondary metabolites from folk medicines [15,17] that involved an Agilent 1260 HPLC system (Agilent Technologies, Santa Clara, CA, USA). A Kinetex C18 column (150 × 4.6 mm; 5 µm) with a C18 guard column (4.00 × 3.00 mm; Phenomenex, Torrance, CA, USA) was used to process the sword bean extract. The mobile phase consisted of solvent A (water with 0.1% formic acid, *v*/*v*) and solvent B (acetonitrile with 0.1% formic acid, *v*/*v*). The chromatography process utilized a flow rate of 0.6 mL/min, an injection volume of 10.0 µL, and an initial mobile phase composition of 15% B for the first 5 min, followed by a linear gradient to 90% B over the next 25 min. The entire operation, including data acquisition and analysis, was managed using Agilent HPLC-Q-TOF-MS MassHunter Acquisition Software (Qualitative analysis, version B.04.00 Build 4.0.479.0).

### 2.3. Plant Material

The sword bean (*C. gladiata*) pods were obtained from Harin Food Co. (Gwangju, Republic of Korea) and identified by Dr. Ki Yong Lee, a professor at the College of Pharmacy, Korea University. A voucher specimen (KUP-HD107) was deposited at the Laboratory of Pharmacognosy, College of Pharmacy, Korea University.

### 2.4. Extraction and Isolation

*C. gladiata* pods weighing 830 g were subjected to methanol (MeOH) extraction (3 × 2 L) at room temperature using the sonication method for 4 h at 25 °C. Concentrating the methanol solution using a rotary evaporator yielded a MeOH extract residue (112.0 g). This residue was further suspended in water (H_2_O) and partitioned successively with *n*-hexane, ethyl acetate (EtOAc), and *n*-butanol (BuOH), resulting in *n*-hexane (3.2 g), EtOAc (2.4 g), BuOH (7.3 g), and aqueous fractions (W). The BuOH fraction was separated using RP-C18 silica gel CC and eluted with a gradient solvent mixture of MeOH-H_2_O (20:80, 40:60, 60:40, 20:80, *v*/*v*, and pure MeOH, stepwise), yielding five fractions (BuOH-1 to BuOH-5). BuOH-2 (500 mg) was further purified using a Sephadex™ LH-20 column and HPLC (MeOH/H_2_O 30/70) for 20 min, resulting in the isolation of compounds **8** (2.5 mg), **9** (2.6 mg), and **10** (10.0 mg). BuOH-3 (1 g) was also purified using a Sephadex™ LH-20 column and HPLC (MeOH/H_2_O 30/70) for 20 min, leading to the isolation of compound **11** (10.0 mg). The EtOAc fraction (2.4 g) was divided into eight smaller fractions, labeled E1–E8, through silica gel CC using *n*-hexane/EtOAc elution (from 50/1 to 0/1, *v*/*v*). Fraction E3 (520 mg) was further subjected to silica gel CC with n-hexane/EtOAc (from 30/1 to 1/1, *v*/*v*) and purified via RP C18 CC with a solvent mixture of ACN/H_2_O (4:1, *v*/*v*), resulting in the isolation of compounds **5** (2.5 mg), **6** (11.7 mg), and **7** (0.3 mg). Similarly, the *n*-hexane fraction (3.2 g) was isolated using silica gel CC and eluted with *n*-hexane/EtOAc (from 50/1 to 1/1, *v*/*v*), yielding 11 smaller subfractions (H-1 to H-11). Subfraction H3 (100 mg) was purified through RP C18 CC using an eluent of acetone–H_2_O (4:1, *v*/*v*), leading to the isolation of compounds **1** (8.0 mg) and **2** (50.5 mg). Finally, subfraction H5 (350 mg) was separated via YMC CC using a MeOH–H_2_O eluent (1/1, *v*/*v*) and further purified by silica gel CC with *n*-hexane/EtOAc (from 50/1 to 1/1, *v*/*v*), resulting in the isolation of compounds **3** (36.0 mg) and **4** (2.0 mg).

### 2.5. Physical and Spectroscopic Data of Bioactive Compounds

Compound **7**. White powder. C_16_H_12_O_6_. HR-ESI-MS *m*/*z* 299.0560 [M-H]^−^ (calculated 299.0561); ^1^H-NMR (600 MHz, DMSO) *δ*_H_: 8.18 (1H, s, H-2), 6.23 (1H, d, *J* = 2.1 Hz, H-6), 6.39 (1H, d, *J* = 2.1 Hz, H-8), 6.47 (1H, d, *J* = 2.5 Hz, H-3′), 6.44 (1H, dd, *J* = 2.5, 8.3 Hz, H-5′), 7.10 (1H, d, *J* = 8.3 Hz, H-6′), 3.72 (3H, s, 7-OCH_3_), 13.20, (1H, br s, 5-OH); ^13^C-NMR (150 MHz, DMSO) *δ*_C_: 155.4, 120.2, 180.3, 161.9, 98.9, 164.2, 93.7, 160.4, 104.5, 110.4, 156.5, 101.4, 157.6, 104.5, 132.3, 55.0 (7-OCH_3_). The NMR data agree with this reference [18].

Compound **10**. Yellow powder. C_27_H_30_O_16_. HR-ESI-MS *m*/*z* 609.1463 [M-H]^−^ (calculated 609.1461). ^1^H-NMR (600 MHz, CD_3_OD) *δ*_H_: 6.20 (1H, d, *J* = 2.0 Hz, H-6), 6.41 (1H, d, *J* = 2.0 Hz, H-8), 7.48 (1H, d, *J* = 2.0 Hz, H-2′), 6.76 (1H, d, *J* = 8.0 Hz, H-5′), 7.68 (1H, dd, *J* = 8.0, 2.0 Hz, H-6′), 5.06 (1H, d, *J* = 7.5 Hz, H-1″), 3.96 (1H, d, *J* = 1.0 Hz, H-1′′′), 1.11 (3H, d, *J* = 6 Hz, H-6′′′). ^13^C-NMR (150 MHz, CD_3_OD) *δ*_C_: 158.8, 135.5, 179.4, 163.2, 100.6, 167.8, 95.6, 159.3, 105.2, 123.2, 116.3, 150.2, 146.3, 117.8, 123.8, 102.6, 75.8, 78.3, 71.4, 77.3, 68.6, 105.2, 72.3, 72.4, 73.8, 69.8, 18.0. The NMR data agree with this reference [15].

Compound **11**. A dark-yellow amorphous powder; C_27_H_30_O_17_. HR-ESI-MS *m*/*z* 795.1951 [M+Na]^+^ (calculated. 795.1960). ^1^H-NMR (600 MHz, CD_3_OD) *δ*_H_ 6.18 (d, *J* = 2.2, H-6) 6.36 (d, *J* = 2.2, H-8) 7.23 (s, H-2′) 7.23 (s, H-6′) 5.55 (d, *J* = 7.8, H-1) 5.21 (d, *J* = 1.6, H-2″) 1.03 (d, *J* = 6.3, H-6″); ^13^C-NMR (150 MHz, CD_3_OD) *δ*_C_: 158.8, 135.6 179.3, 163.1, 99.8, 165.6, 95.6, 158.4, 105.9, 123.5, 110.2, 146.4, 137.9, 146.4, 110.2, 100.6, 80.4, 78.9, 71.8, 77.1, 68.4, 105.1,72.1, 72.1, 73.9, 69.7, 17.9. The NMR data agree with this reference [19].

### 2.6. IL-33 Protein Expression and Purification

The expression and purification of the IL-33 protein were carried out similarly to a previous report with slight modifications [17]. In brief, IL-33 (117-270) with an N-terminal His-tagged fusion was cloned into the expression vector pPROEX (DE3) and introduced into Escherichia coli BL21. Cell cultures were grown at 20 °C and induced with 0.5 mM isopropylthio-β-galactoside when the optical density at 600 nm reached 0.6. For the production of uniformly labeled 15 N IL-33 (117-270), bacterial cells were cultured in M9 minimal medium supplemented with 15 N NH4Cl. Subsequently, the cells were harvested, resuspended in a lysis buffer (0.1 M Tris-HCl, pH 7.4, 0.3 M NaCl, 1 mM mercaptoethanol, 0.1% Triton X100, and 0.1 mM phenylmethylsulfonyl fluoride), and sonicated in an ice-cold bath to disrupt the cells. The pure IL-33 protein was obtained through Superdex S75 gel filtration chromatography (16/60; GE Healthcare, Chicago, IL, USA) using 20 mM Na phosphate buffer (pH 6.8), 100 mM NaCl, and 5 mM basement membrane extract.

### 2.7. The Enzyme-Linked Immunosorbent Assay Technique

The ELISA test was conducted following a protocol similar to that described in a previous report, with minor adjustments [17]. In this setup, the HIS ST2 to wild-type ST2 ratio was adjusted as follows: 20:80 for 80% inhibition, 40:60 for 60% inhibition, 60:40 for 40% inhibition, 80:20 for 20% inhibition, and 100:0 for non-inhibition. Each well underwent three rounds of washing with 220 µL of phosphate-buffered saline with Tween-20 (PBST). After the washes, each well was exposed to 100 µL of a diluted horseradish peroxidase antibody (Abcam, Cambridge, UK) in PBST and then incubated for 1 h. Following incubation, 100 µL of tetramethylbenzidine liquid substrate (Thermo Fisher Scientific, Waltham, MA, USA) was added to each well after three PBST (220 µL) washes. Each well was incubated for 10 min, and 1 M HCl was added to stop the reaction. The optical density was measured using a microplate spectrophotometer set at 450 nm. The degree of IL-33 inhibition was determined based on a calibration trend line.

### 2.8. Molecular Docking Simulation

The molecular docking simulation was conducted following a protocol similar to that described in the previous report, with slight modifications [20]. We obtained the crystal structure of the IL-33 and ST2 receptor complex (PDB-ID: 4KC3) from the Protein Data Bank (http://www.rcsb.org (accessed on 16 October 2023)) for use in the in silico study [20]. The protein’s conformation was optimized using the “Orthogonal Partial Least Squares 4” (OPLS4) potential energy function and the protein preparation tool in Maestro v12.4 (Schrodinger, New York, NY, USA) until the average RMSD of non-hydrogen atoms reached 0.3 Å. We used ChemDraw software 20.0 (PerkinElmer Informatics, Waltham, MA, USA) to create 2D structures of 13 ligands. These 2D structures were then converted into 3D structures using the LigPrep tool (Schrodinger), resulting in molecular structures optimized at pH 7.0 ± 2.0. The 3D structures of the ligands revealed their chirality, and the energy of the 3D conformers was minimized using OPLS4 as the final step in LigPrep. For receptor grid generation, we utilized the Receptor Grid Generation tool in Maestro v12.4 (NM share, version 6.0.134; Schrödinger) to define the docking grid for the receptor’s active site based on the coordinates from the PDB file. The grid was generated at the position of the co-crystallized ligand. The active site’s region, characterized by X, Y, and Z coordinates, was used to define this site. The X, Y, and Z axes corresponded to coordinates 38.31, −39.24, and 12.96, respectively, which marked the center of the receptor grid box. The dimensions of the grid box were adjusted to match the size of the co-crystallized ligand. Glide was employed in extra precision (XP) mode for docking and computations.

### 2.9. Molecular Dynamic Simulation

Similar to other reports [21], we conducted a molecular dynamics simulation to gain a better understanding of the interactions in the protein–ligand complex. The complex was prepared using the system builder tool. The solvent model employed was the simple point charge model used to mimic water molecules. The binding site of the protein–ligand complex was placed in an orthorhombic box with a distance of 10 Å in all directions and then minimized for volume. To neutralize the entire system, Na+ ions were added, and the salt concentration was set to 0.15 M using Na+ as the positive ion and Cl− as the negative ion. The OPLS4 force field was chosen to minimize the energy of the complex. Molecular dynamics simulations were carried out with the isothermal–isobaric (NPT) ensemble at 300 K and 1.01325 bars. Temperature and pressure were maintained using the Nosé–Hoover chain thermostat method and the Martyna–Tobias–Klein barostat method, respectively. The simulation ran for 50 ns with a recording interval of 50 ps. The entire model system was equilibrated before the simulation. The results of the molecular dynamics simulation were analyzed using the simulation interaction diagram tool in Maestro v12.4.

### 2.10. Statistical Analyses

Statistical analysis was performed using GraphPad Prism 5 software version 5 (San Diego, CA, USA). The results are presented as mean ± standard deviation of three independent experiments. Statistical significance was determined at *p* < 0.05.

## 3. Results and Discussion

### 3.1. Screening Secondary Metabolites Using Liquid Chromatography Quadrupole Time-of-Flight Mass Spectrometry Conditions for Target Isolation

**Figure 1:**
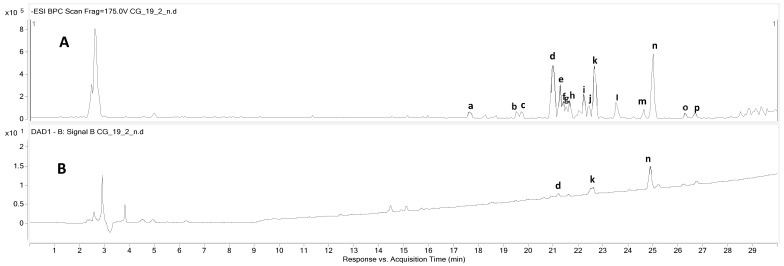
To screen secondary metabolites from the MeOH extract of *C. gladiate*, LC-QTOF MS/MS was conducted in negative mode. (**A**) Negative mode mass (MS) chromatogram. (**B**) Ultraviolet (UV; 254 nm) chromatogram of the methanol extract from *C. gladiata*.

The secondary metabolites of *C. gladiata* in the methanol extract were assessed under liquid chromatography–mass spectrometry (LC-MS) guidance. Several major compounds were identified by comparing their retention times, MS/MS fragments, maximum ultraviolet (UV) absorption (hereinafter, UV max), molecular weight, and references. (Figure 1). Specifically, the following phenolic compounds were detected for peaks **c**, **e**, **h**, **j**, **k**, **m**, **n**, and **o**: hydroxybenzoic acid, genistein, methoxy kaempferol, pseudobaptigenin, formononetin, 5′-hydroxy pseudobaptigenin, biochanin A, and sophorophenolone, respectively. The chemical profiling of methanol extract is detailed in Table 1. Once the target compounds were identified, we applied isolation techniques to separate and purify them.

### 3.2. Extraction and Purification of Bioactive Compounds from C. gladiata

**Figure 2:**
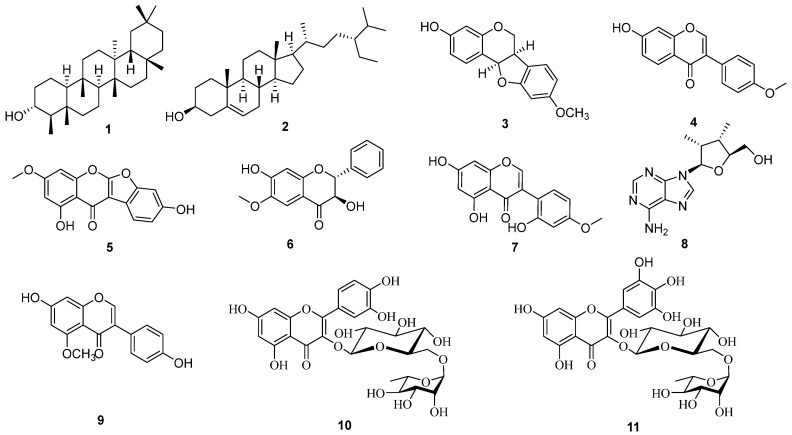
Structures of compounds **1**–**11** isolated from *C. gladiata*. The structures were generated using ChemDraw software version 20.0 (PerkinElmer Informatics, Waltham, MA, USA).

The methanolic extract from *C. gladiate* pods was fractionated utilizing organic solvents of increasing polarity: *n*-hexane, ethyl acetate, aqueous *n*-butanol, and the water layer, respectively. Eleven compounds (**1**–**11**) were separated through repeated column chromatography (CC) using silica gel, RP-18, and semi-preparative high-performance liquid chromatography (HPLC), as shown in Figure 2. Through a comparison of their spectroscopic data with the literature references using DeepSAT (https://deepsat.ucsd.edu/ (accessed on 30 December 2021)) [22], the chemical structure was identified as 3*α*-friedelinol (**1**) [23], *β*-sitosterol (**2**) [24], medicarpin (**3**) [25], formononetin (**4**) [26], sophorophenolone (**5**) [27], 7-hydroxy-6-methoxy dihydroflavonol (**6**), 2′-hydroxy biochanin A (**7**) [28], *β*-adenosine (**8**) [29], 5-methoxydaidzein (**9**) [30], rutin (**10**) [19], and myricetin 3-*O*-rutinoside (**11**) [31]. To our knowledge, this study is the first to document compounds **1**, **2**, **4**, and **11** in sword bean (*C. gladiata*). Consequently, our research enhances the understanding of secondary metabolites and offers a scientific basis for the utilization of sword bean. However, this study only identifies the chemical components of the Korean sword bean. Further research is needed to investigate the secondary metabolites of the sword bean collected from various locations and seasons. This deeper evaluation of chemical profiling will help in exploring potential industrial applications. Furthermore, future studies should address additional optimization conditions for the extraction process or explore alternative extraction methods.

### 3.3. In Vitro Evaluation of IL-33/ST2 Interaction with Isolated Compounds Using ELISA

**Figure 3:**
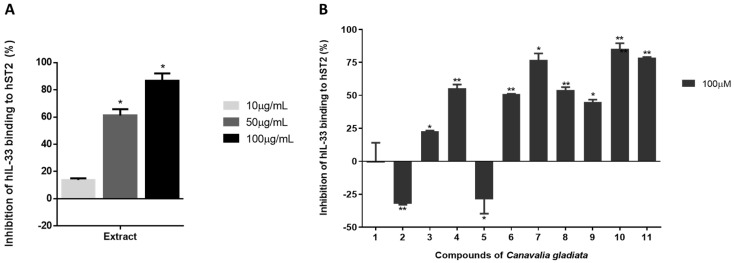
Assessing the inhibitory effect of extract and isolated compounds on IL-33/ST2 interaction using ELISA (enzyme-linked immunosorbent assay). ELISA results showing the repression of IL-33/ST2 interaction in (**A**) *C. gladiata* MeOH extract (10, 50, and 100 µg/mL). (**B**) Isolated compounds, **1**–**11** (100 µM). * *p* < 0.05, ** *p* < 0.01.

As highlighted in the Introduction section, the critical regulatory functions and involvement in diverse metabolic pathways make both IL-33 and its receptor ST2 promising targets for treating allergies and cancer. Evaluating the interaction between IL-33 and ST2 is crucial because ST2 is a part of the receptor for IL-33, known as the IL-33 receptor (IL-33R). When IL-33 binds to ST2, it triggers biological responses, including immune cell stimulation and inflammation [21]. Assessing the interaction between IL-33 and ST2 can provide vital information about the inhibitory potential of compounds in this system, aiding in evaluating their potential for treating inflammation and immune-related diseases. To evaluate the potential inhibitory effect of the compounds on the IL-33/ST2 interaction, we employed ELISA [17]. Briefly, microplate wells were coated with ST2 protein as the capture antigen. Then, IL-33 protein, acting as the target antigen, was added to the wells to bind with the immobilized ST2. Subsequently, various concentrations of the test compounds were introduced to the wells to interact with the IL-33/ST2 complex. To evaluate the pharmacological properties of *C. gladiata*, we examined the purified compounds (**1**–**11**) for their impact on IL-33/ST2 using the enzyme-linked immunosorbent assay (ELISA) method (Figure 3). The results reveal that compounds **4** and **6**–**11** exhibited significant IL-33/ST2 inhibitory activity. Notably, compounds **7**, **10**, and **11** demonstrated substantial IL-33/ST2 inhibitory efficacy, reaching 78%, 86%, and 79% at 100 µM, respectively. Consequently, compounds **7**, **10**, and **11** were considered as potential therapeutic agents for allergies.

**Figure 4:**
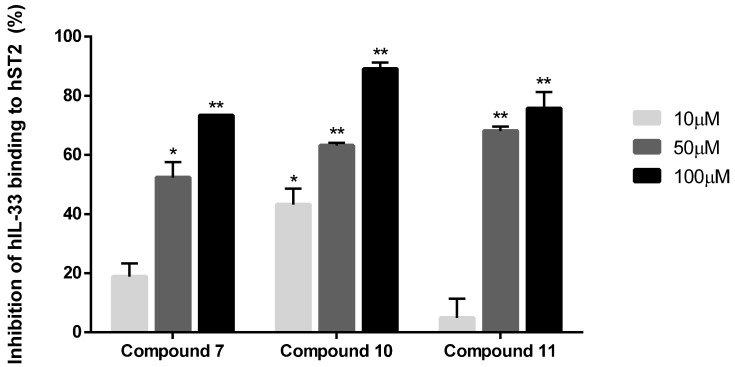
Evidence of the dose-dependent effects of compounds **7**, **10**, and **11** on IL-33 protein. * *p* < 0.05, ** *p* < 0.01.

According to the isolation process, two flavonoid glycosides, compounds **10** and **11**, were obtained at 10 mg each. This deduction suggests that compounds **10** and **11** are the main components of *C. gladiata*. Based on the screening results, substances **7**, **10**, and **11** were chosen for further exploration due to their remarkable IL-33/ST2 suppression. Isoflavonoid **7**, along with two flavonoid glycosides, **10** and **11**, demonstrated inhibition that increased with concentration (10, 50, and 100 µM). Therefore, compounds **7**, **10**, and **11** exhibited concentration-dependent inhibition (Figure 4). These findings suggest that the Korean sword bean’s secondary metabolites may have beneficial pharmacological (anti-allergic) properties.

The potential of numerous medications to inhibit IL-33 has been examined [32], and research into the use of anti-IL-33 antibodies for managing conditions such as asthma and allergic rhinitis is ongoing. Novel treatments, including various “IL-33 traps” with complex ST2 receptors and IL-1RAcP coreceptors, have been developed to neutralize IL-33, leading to a reduction in airway inflammation in allergies. Notably, a recently discovered protein called HpARI, secreted by a mouse helminth parasite, has been shown to inhibit IL-33 activity, thereby lowering type 2 immune responses [33].

### 3.4. In Silico Evaluation of Pharmacological Effects

#### 3.4.1. Molecular Docking Simulation

While there have been numerous studies on IL-33/ST2 protein inhibition, medical research and development of new medications for allergies and IL-33/ST2 inhibition are ongoing. In a previous study, we reported the potential inhibition of IL-33 by triterpenoid saponins isolated from *Astragalus membranaceus* [17]. Both asthma and allergic rhinitis have been the focus of research on anti-IL-33 antibodies. Researchers are developing small chemical inhibitors to disrupt the interaction between IL-33 and the ST2 receptor, offering a unique approach to target the IL-33/ST2 interface. In a recent study [34], various small compounds that bind to IL-33 were discovered by investigating a second, flexible binding site for the ST2 receptor. We identified compounds **7**, **10**, and **11** as disruptors of the IL-33 and ST2 interaction in our in vitro study. Molecular docking simulations are widely used in structure-based drug discovery. They provide atomic-level insights into the intricate interactions between proteins and small molecules, shedding light on vital biochemical processes at specific binding sites on target proteins.

Virtual screening is a flexible and cost-effective method that facilitates the search for suitable protein inhibitors. In this study, we employed compounds **10** and **11**, as they demonstrated potential in binding to IL-33. The crystal structure of the IL-33 protein, in association with 2-acetamido-2-deoxy-β-D-glucopyranose (PDB ID: 4KC3), informed our identification of the drugs’ active regions, critical interactions, and molecular mechanisms related to IL-33 protein inhibition.

**Figure 5:**
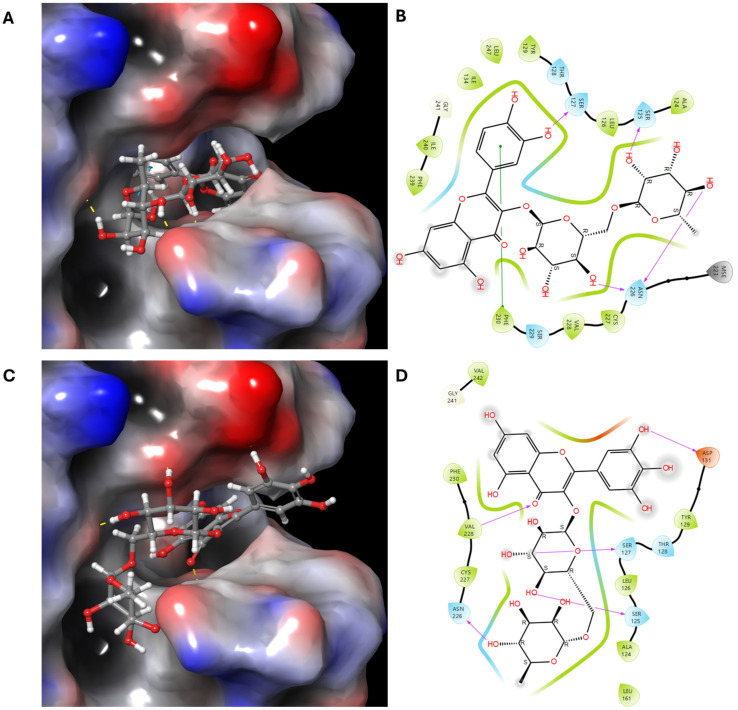
The crystal structure of the IL-33 and ST2 receptor complex (PDB ID: 4KC3) was obtained from the Protein Data Bank (http://www.rcsb.org (accessed on 20 October 2023)) for use in the molecular docking simulation. The protein’s conformation was optimized using the ‘Orthogonal Partial Least Squares 4’ (OPLS4) potential energy function and the protein preparation tool in Maestro v12.4 (Schrödinger, New York, NY, USA). Molecular docking for compounds **10** and **11** with 4KC3 protein: (**A**) Three-dimensional (3D) and (**B**) Two-dimensional (2D) docking simulation of the interactions between compound **10**. (**C**) Three-dimensional (3D) and (**D**) Two-dimensional (2D) docking simulations of the interactions between compound **11**. Compounds 10 and 11 have transparent surfaces on their ligand-binding pockets (left panels). The right panels depict interactions between the ligands and the protein’s amino acid residues, such as hydrogen bonds (pink) and pi–pi stacking interactions (green).

Compounds **10** and **11** exhibited strong binding to the catalytic site of IL-33, with binding affinities of −12.383 and −11.901 kcal/mol, respectively (Table 2), corroborated by in vitro experiments. Based on the high binding affinities of both compounds, **10** and **11**, it is inferred that they exhibit strong interactions with the target 4KC3 protein and may hold therapeutic potential. Further investigation as potential drug candidates for diseases related to the inhibition of the IL-33/ST2 pathway is warranted. Compound **10** appeared to be associated with the IL-33 interaction interface, as depicted in Figure 5. It established strong hydrogen bonding interactions with multiple amino acid residues, including SER125 (2.73), SER127 (2.06), and ASN226 (2.07, 2.65), within IL-33’s active site. Compound **11** formed hydrogen bonds with SER125 (2.45), SER127 (2.09), ASP131 (1.88), ASN226 (1.88), and VAL228 (2.52) in IL-33’s active site. Compounds **10** and **11** both demonstrated potent inhibitory activity against the IL-33 protein. When considering the results of the molecular docking studies and ELISA, these compounds hold promise as natural treatments for allergic conditions.

#### 3.4.2. Molecular Dynamic Simulation

**Figure 6:**
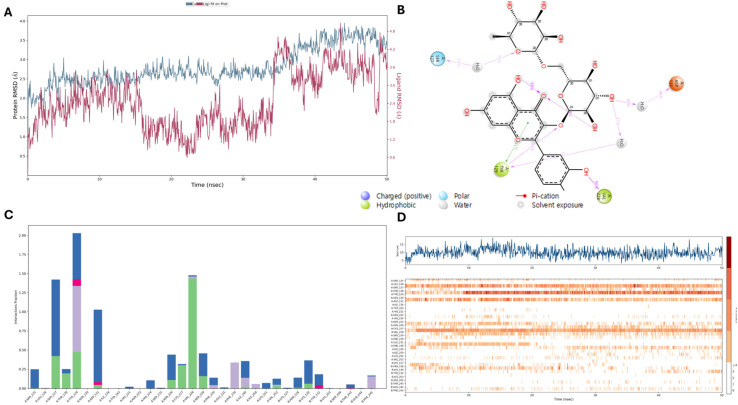
Molecular dynamics simulation results of compound **10** interacting with the IL-33 protein. (**A**) For both the ligand (red) and the Cα backbone (blue), the root mean square deviation plot demonstrated steady convergence over 50 ns. (**B**) A 2D illustration of the interaction between substance **10** and 4KC3. (**C**) The protein–ligand affinities were examined. (**D**) A chronological illustration of the evolution of these contacts over a 100 ns simulation period.

Molecular dynamics is a method used to determine the dynamic state of a ligand–receptor complex. Unlike molecular docking, it offers comprehensive insights into ligand–receptor interactions. We employed molecular dynamics simulations to assess the stability of the IL-33-flavonoid glycoside complex. A 50 ns simulation indicated consistent conformational stability; root mean square deviation (RMSD) analysis of the Cα atoms showed that the complex became stable at around 25 ns and remained so thereafter. This suggests that following a short period of initial fluctuation, the 4KC3 protein–ligand complex attained a stable state and remained largely unchanged throughout the simulation. This can be interpreted as the complex achieving structural stability, reflecting a balance between internal and external forces within the system. The protein Cα-backbone bound to **10** exhibited an RMSD deviation of 3.2 Å, whereas the ligand’s Cα-backbone showed a deviation of 2.5 Å (Figure 6). The similarity in RMSDs of both the protein and ligand confirmed the complex’s stability. An RMSD plot during the simulation demonstrated strong convergence and stable conformations within an acceptable range. These values indicated sustained stability in the receptor–ligand complex throughout the simulation. The remarkable stability of the complex between **10** and the protein was primarily due to the ligand’s stronger affinity. Figure 6B displays the two-dimensional (2D) interactions of **10** with binding cavity residues, showing conventional hydrogen bonding with TYR129 and VAL228; H_2_O bridge formation with SER127, TYR129, and ASP131; and hydrophobic interactions with TYR129. Notably, TYR129 exhibited all of these interactions, and VAL228 maintained a hydrogen bond with the protein approximately 96% of the time during the simulation. These interactions remained consistent throughout the experiment (Figure 6C,D). The examination of the ligand’s interactions with the binding cavity residues unveiled diverse interaction types, encompassing conventional hydrogen bonding, H_2_O bridge formation, and hydrophobic interactions. Notably, TYR129 participated in multiple interactions, underscoring its significance in stabilizing the complex. Moreover, VAL228 sustained a hydrogen bond with the protein for a considerable duration of the simulation, bolstering the complex’s stability. Consequently, our in silico experiments provided evidence for the anti-allergic properties of flavonoid glycosides from sword beans, consistent with our in vitro findings.

## 4. Conclusions

Recognizing the inherent limitations of in silico methods in fully elucidating the mechanism of action, it is imperative to conduct future in vivo studies. These studies will play a pivotal role in uncovering the therapeutic potential of compound **10** as an inhibitor of the IL-33/ST2 signaling pathway for the treatment of ADs. Natural products derived from medicinal herbs play a pivotal role in advancing the field of pharmaceutical research [16]. Notably, over the past three decades, approximately 60% of low-molecular-weight medications approved by the Food and Drug Administration have either been derived directly from natural sources or have strong connections to them [35]. The sword bean (*C. gladiata*) is a common domestic vegetable in Asian countries, and is also used in Chinese Oriental medicine. Sword beans show a wide range of pharmacological effects, including antioxidative, antibacterial, anti-gastric inflammatory, and antiangiogenic activities, and they have the potential to alleviate digestive disorders. To the best of our knowledge, there has been limited research on the secondary metabolites from the sword bean. Additionally, its potential anti-allergic effects have not been reported previously. In this research, a chemical analysis of a methanol extract from the Korean sword bean led to the identification of 11 compounds (**1**–**11**) through the integration of LC-Q-TOF MS with a bioassay-directed method. Compounds **10** and **11** were identified as rutin and myricetin 3-*O*-rutinoside, respectively, based on the combined chromatography separation techniques and HPLC analysis. Interestingly, compounds **4** and **6**–**11** exhibited significant inhibitory activity at 100 µM. Flavonoid glycosides **10** and **11** demonstrated inhibition with increasing concentrations (10, 50, and 100 µM). Additionally, an in vitro and in silico analysis revealed that compounds **10** and **11** exhibited significant potential as suppressor compounds in autoimmune diseases (ADs) by modulating the IL-33/ST2 signaling pathway. This study has identified several bioactive compounds from the Korean sword bean that target the inhibition of the IL-33/ST2 signaling pathway. The study’s limitation is that it solely relies on in vitro and in silico methods for evaluation, lacking validation from in vivo studies. Consequently, it is advisable to conduct further analyses and in vivo studies to gain a better understanding of the precise mechanisms of action of these compounds. As a result, these compounds should be considered for further investigation, including in vivo research, to better understand their precise mechanisms of action. Several in vitro concentrations showing potential pharmacological effects up to 100 µM have been reported thus far [17,36]. Nevertheless, meticulous attention should be paid to the concentrations of active compounds to ensure the feasibility of in vivo studies aligned with pharmaceutical design. The current study showed that flavonoid glycosides **10** and **11** from the Korean sword bean may have therapeutic potential for ADs.

## Figures and Tables

**Table 1:** Screening for bioactive compounds from *C. gladiata* using LC-QTOF MS/MS.

Peak No.	Expected Compounds	t_R_ (min)	Observed *m*/*z*	Calculated *m*/*z*	Molecular Formula [M-H]^−^	MS/MS Fragments (*m*/*z*)	UV (λmax, nm)
**a**	Unidentified	17.656	331.0809	331.0823	C_17_H_15_O_7_	288 [M-43-H]^−^	
**b**	Unidentified	19.588	283.0598	283.0612	C_16_H_11_O_5_	268 [M-CH_3_-H]^−^	
**c**	Hydroxybenzoic acid	19.716	137.0235	137.0244	C_7_H_5_O_3_	93 [M-CO_2_-H]^−^	
**d**	Unidentified	20.965	285.0757	285.0768	C_16_H_13_O_5_	270 [M-CH_3_-H]^−^	281, 339
**e**	Genistein	21.278	269.0442	269.0455	C_15_H_9_O_5_	133 [M-C_7_H_4_O_3_-H]^−^	285, 350
**f**	Unidentified	21.402	299.0545	299.0561	C_16_H_11_O_6_	284 [M-CH_3_-H]^−^	
**g**	Unidentified	21.527	301.0703	301.0718	C_16_H_13_O_6_	165 [M-136-H]^−^	
**h**	Methoxykaempferol	21.652	299.0547	299.0561	C_16_H_11_O_6_	-	
**i**	Unidentified	22.214	301.0704	301.0718	C_16_H_13_O_6_	125 [M-176-H]^−^	
**j**	Pseudobaptigenin	22.464	281.0443	281.0455	C_16_H_9_O_5_	253 [M-C_2_H_4_-H]^−^	
**k**	Formonetin	22.651	267.0651	267.0663	C_16_H_11_O_4_	252 [M-CH_3_-H]^−^	284, 350
**l**	Unidentified	23.525	193.0854	193.0870	C_11_H_13_O_3_	177 [M-16-H]^−^	
**m**	5′-Hydroxy pseudobaptigenin	24.649	297.0386	297.0405	C_16_H_9_O_6_	269 [M-C_2_H_4_-H]^−^	
**n**	Biochanin A	25.024	283.0593	283.0612	C_16_H_11_O_5_	268 [M-CH_3_-H]^−^	260
**o**	Sophorophenolone	26.273	297.0388	297.0405	C_16_H_9_O_6_	282 [M-CH_3_-H]^−^	
**p**	Unidentified	26.710	194.0808	194.0823	C_10_H_11_NO_3_	-	

**Table 2:** In silico molecular docking simulations of bioactive compounds for therapeutic studies.

Compound	XP Dock Score (kcal/mol)	Hydrogen Bonding Interactions (Å)	Pi–Pi Stacking (Å)
**10**	−12.383	SER125 (2.73), SER127 (2.06), ASN226 (2.07, 2.65)	PHE230 (5.42)
**11**	−11.901	SER125 (2.45), SER127 (2.09), ASP131 (1.88), ASN226 (1.88), VAL228 (2.52)	-

## Data Availability

Data is contained within the article.
